# Ethics experts and fetal patients: a proposal for modesty

**DOI:** 10.1186/s12910-021-00730-3

**Published:** 2021-12-03

**Authors:** Dagmar Schmitz, Angus Clarke

**Affiliations:** 1 Institute for History, Theory and Ethics in Medicine, RWTH Aachen University, Wendlingweg 2, 52074 Aachen, Germany; 2 School of Medicine, Institute of Medical Genetics, Cardiff University, Wales, UK

**Keywords:** Ethics consultation, Prenatal medicine, Professional ethics

## Abstract

**Background:**

Ethics consultation is recognized as an opportunity to share responsibility for difficult decisions in prenatal medicine, where moral intuitions are often unable to lead to a settled decision. It remains unclear, however, if the general standards of ethics consultation are applicable to the very particular setting of pregnancy.

**Main text:**

We sought to analyze the special nature of disagreements, conflicts and value uncertainties in prenatal medicine as well as the ways in which an ethics consultation service (ECS) could possibly respond to them and illustrated our results with a case example. Ethics facilitation and conflict mediation, currently, have no broadly consented normative framework encompassing prenatal diagnosis and therapy as well as reproductive choice to draw on. Even so, they can still be helpful instruments for ethically challenging decision-making in prenatal medicine provided two additional rules are respected: For the time being, ECSs should (a) refrain from issuing content-heavy recommendations in prenatal medicine and (b) should not initiate conflict mediations that would involve the pregnant woman or couple as a conflict party.

**Conclusion:**

It seems to be vital that ethics consultants as well as health care professionals acknowledge the current limitations and pitfalls of ethics consultation in prenatal medicine and together engage in the advancement of standards for this particularly complex setting.

## Background

### Ethics consultation in pregnancy

The clinical practice of prenatal, maternal–fetal medicine is characterized by a variety of specific ethical challenges. They are raised for all agents involved by not only the possible termination of a pregnancy but also the ever broadening range of available options for prenatal diagnosis and therapy. Not surprisingly, prenatal medicine is a relevant site for ethics consultation services (ECSs). There is little information available on how often and by whom ethics consultation is requested in this context. However, there is at least anecdotal evidence from single center studies that issues relating to pregnancy, and especially requests for a termination of pregnancy, regularly trigger ethics referrals [1–4]. In some hospitals, ECSs are required to participate in every decision-making process concerning a late termination of pregnancy [5] or have been established specifically for the context of prenatal testing and selective terminations of pregnancy [6, 7]. Ethics consultation seems to be recognized as an opportunity to share responsibility for difficult decisions in the prenatal context, where moral intuitions are often unable to lead to a settled decision. But is pregnancy just another field of action for ethics consultation services, comparable to the end of life or to psychiatric diseases? Are the general standards of ethics consultation applicable to the very particular setting of pregnancy? What can clinicians expect from ethics consultation in prenatal medicine?

We will use a case example to introduce the ethical peculiarities of prenatal medicine and the ways, an ECS working with a facilitation approach can respond.

Case example

An ECS was asked by a consultant neonatologist (brought in by fetal medicine) to discuss the case of a woman whose fetus had been found on scan to suffer from multiple malformations, thought likely to be the result of a chromosomal abnormality such as an autosomal trisomy. The malformations were severe and were considered to be incompatible with long-term survival, with the affected infant perhaps likely to survive for some hours or at most a few days.

The neonatologist had referred the case to the ECS as she wished an amniocentesis to be performed on the pregnancy, then at 24 weeks’ gestation, expecting to find a cytogenetic diagnosis. This would allow her team, she said, not to resuscitate the infant at birth but offer supportive care only. The parents, on considering their baby’s condition, had made clear their wish that the baby be born naturally and then nursed but not resuscitated intensively. The clinical team had put it to the pregnant woman that they would feel compelled to impose intensive resuscitation at birth unless she agreed to have an amniocentesis. It was hoped that the referral to the ECS would resolve this dispute between the neonatal team and the pregnant woman, with the clinician hoping that it would “persuade” (i.e. add to the pressure on) the couple to agree to the amniocentesis, which might or might not have led to a chromosomal diagnosis in the fetus. Underlying the referral was the neonatologist’s concern that medical and/or nursing staff would insist on inevitably futile attempts at full resuscitation of the dying infant without a severe chromosome anomaly being found; amniocentesis was seen as a potential route to avoid that outcome, unsought by anyone.

## The method of ethics consultation

ECSs are delivered by committee members, a group of or single consultants who take action in response to requests for assistance [8, 9]. The requesting parties (health care professionals, patients, relatives) may have experienced uncertainties or conflict in decision-making processes in clinical practice, concerning their sense of values or norms.

In our case example, a disagreement with the couple eventually brought the team to involve the ECS. The team and the couple disagreed on the value of an invasive procedure (amniocentesis) in the actual clinical situation from an ethical point of view.

In such a situation an ECS is expected to “improve the quality of health care through identification, analysis, and resolution of ethical questions or concerns” [8]. However, there is still a substantial dissent not only about the method but also about the ends that ethics consultation should pursue [10] and who should be performing this service [11]. The theory and practice of ethics consultation is still characterized by a „great divide” [12] between two different models, the clinical (consultation) model and the facilitation/mediation model. The first ethics consultants started 40 years ago, when they delivered verdicts, gave advice or recommended certain courses of action [13]. In doing so, they acted more like “a clinical professional who possesses a specific expertise” and on that basis gave advice about the ethically best course of action or the solution of a conflict [14]. The more recent ethics facilitation approach, in contrast, which is recommended by the American Society for Bioethics and Humanities as an “appropriate approach to ethics consultation” [8], aims at working towards a “principled ethical resolution” [15] in case of conflict or uncertainty regarding values or norms that emerge in health care. The role of the ECS here is much more characterized by the skills required to support others (health care professionals as well as patients and relatives) in decision-making and to mediate between the parties in a conflict. Giving recommendations is not precluded [8, 15, 16], but it is not the first and foremost duty of the ECS. We choose to concentrate on the recommended ethics facilitation approach when describing the processes and challenges of ethics consultation in prenatal medicine in the following chapters. Part of our work is also relevant for the applicability of the clinical (consultation) approach in prenatal medicine.

In ethics facilitation, an ECS supports the requesting parties mainly in three aspects [8, 15]: (a) understanding the ethical nature of the value uncertainty or conflict (including the presented and latent interests of all conflict parties [15]), (b) defining the range of ethically acceptable solutions and (c) resolving the conflict.

### Understanding: The special nature of value uncertainties and conflicts in prenatal medicine

In prenatal medicine, an ECS has to deal with special circumstances. A pregnancy is (1) not per se a state of disease and the typical role concepts for physician–patient interactions frequently do not apply because of the differing ends of clinical practices in prenatal medicine. There is (2) not the typical individual patient with whom physicians aim to interact. Clinical actions in prenatal medicine are usually affecting the pregnant woman and the embryo or fetus at the same time. Some prenatal medicine services are directed towards the prevention or early detection of diseases or impaired function in the pregnant woman (that may transform her, sometimes quite rapidly, into a patient). Most services in prenatal or perinatal medicine, however, are not concerned with the pregnant woman’s health so much as with that of the fetus. Some fetal treatment options put the pregnant woman at risk, while trying to benefit the fetus. In addition, “health” is (3) not the only aim of prenatal medicine. In many situations, services are directed towards enabling reproductive choice, which impacts not only the pregnant woman but potentially also the embryo or fetus.

In our case example, the health of the fetus (or rather the health of the future child) is in the center of attention and a cause for concern. The health of the pregnant woman is not a primary interest in the discussion, but indirectly affected by the decision. The neonatologist, being the specialist for the health of the future child, prioritises the supposed interests of the future child in wishing to confirm a diagnosis and the related poor prognosis, before agreeing to palliative treatment. An additional layer concerns the interest of the team to safeguard the medical decision-making (especially when it has life-limiting consequences) and avoid litigation. The possible interests of the pregnant woman regarding herself (avoiding an invasive procedure; perhaps also choosing not to live with a child with such severe malformations) and her child (avoiding any suffering of the child and having palliative care because of the detected malformations, irrespective of the genetic test results), in contrast, were obviously seen as less important. In terms of ethical principles, the discussion in the professional team revolved mainly around beneficence and non-maleficence concerning the fetus and the future child, whereas the (reproductive) autonomy and well-being of the pregnant woman herself seemed to be of minor relevance for the decision at stake.

In sum, prenatal medicine presents a different set of challenges compared to the everyday questions of clinical ethics, for example at the end of life. These challenges are grounded in the special situation of pregnancy with the pregnant woman and the fetus having such strong connections, both physically and emotionally, while at times their fundamental rights or interests may seem to conflict. An ECS as a new and neutral agent can be extremely helpful in sorting out the various presented or latent interests of the conflict parties and understanding their ethical relevance. But what else can clinicians in prenatal medicine expect from an ECS? How can a resolution be facilitated in such a complex situation?

### Defining: A principled resolution?

An important task of ethics facilitation is to define the range of ethically acceptable (“principled”) solutions to the problem. This range is determined by “clearly accepted ethical principles, legal stipulations, and moral rules defined by ethical discourse, legislatures, and courts” [15]. In prenatal medicine, however, it is hard to identify such an universally accepted framework of principles and arguments to draw upon [17]. Instead, the field is characterized by numerous controversial and heated debates about the most fundamental issues such as the moral status of unborn life [18] and questions of justice [19, 20] and discrimination [21] in prenatal testing.

In order to avoid these fundamental debates, efforts have been made to build the normative framework in prenatal medicine as more or less independent of any statements regarding the moral status of the fetus. McCullough and Chervenak began in the 1980s to elaborate on the duties of health care professionals in prenatal medicine [22]. They stated that fetuses can be presented as patients to health care professionals by the pregnant woman, which gives them a dependent moral status accompanied by beneficence-based (not rights-based) obligations of health care professionals. However, the adequacy and applicability of the concept is under considerable debate [23–25]. The authors repeatedly stressed that a fetal patient is neither required to be seen as separate from the pregnant woman, nor does it necessarily include any positioning towards an independent moral status [26]. But for many readers the concept of a patient seems to imply precisely this: separateness and an independent moral status [24]. This is an inherent contradiction within the McCullough and Chervenak framework, which has not yet been resolved. The “pragmatic concept” [26] of a dependent moral status of a fetus, gaining relevance with increasing gestational age, might converge with many moral intuitions of health care professionals and the public, alike. The more problematic step, however, is to base this dependent moral status on the social role of being a patient. Lyerly et al. rightly pointed out that the “*paradigmatic* patient is an entity physically individuated and fully separate from others. It is against this broad backdrop that those in medicine think about examining, diagnosing and treating patients.” [24] Critics fear that the concept of a fetal patient, therefore, is misleading with regard to the moral status of the fetus and to the rights of the pregnant women by supporting a perspective which sees the pregnant woman as an “environment” of the fetal patient,—a mere means and not an end in herself [27–29]. If we were to succeed in relocating the dependent moral status of fetuses in a different conceptual framework (e.g. the rights or interests of the future child)^1^ without at the same time attributing a patient role to the fetus, we might be able to develop a sound normative foundation for prenatal medicine. But, so far, a generally accepted normative concept for physician–patient-interactions in prenatal medicine and the related professional ethos is still missing [28, 30].

The ECS in our case example, accordingly, has no universally accepted guidelines at hand on how to handle conflicting interests of the pregnant woman and the future child in the prenatal situation. For Chervenak and McCullough, the gestational age (viability) and the existence of medical interventions that clearly benefit the fetus is decisive in order to determine if a physician has beneficence-based obligations towards the fetus or future child [22]. But even if such obligations are to be confirmed, they would have to be weighed against beneficence- and autonomy-based obligations towards the pregnant woman. Forcing or persuading the pregnant woman into an amniocentesis would hardly be an ethically acceptable solution based on their account.

A different approach to professional ethics in prenatal medicine with a stronger focus on the pregnant woman and an understanding of fetal interests as an essential part of maternal interests (see for example [28]) might even deny a separate beneficence-based obligation to the fetus and would probably lead to a significantly narrower range of ethically acceptable solutions.

Unsettled is also the relevance of a future child’s informational interests (or rights) for decision-making in prenatal medicine. Especially broad-scope prenatal (genomic) testing has the potential to harm the right (or interest) of a future person not to know their own genetic status and might lead to a conflict with maternal or parental interests [17].

In sum, it seems to be important that an ECS openly communicates the current variability and vagueness of ethical concepts in the field of prenatal medicine and together with clinicians engages in discussing and weighing the different approaches with respect to their relevance for the individual consultation request and its possible solutions. In the absence of a broadly consented normative framework for prenatal medicine, it is hard to see what potentially could be the basis for any content-heavy recommendation or advice of an ECS. Any such statement would not easily be defended against the accusation of arbitrariness and should, therefore, be avoided.

### Defining: The special case of reproductive choice

When prenatal medicine is not concerned with prevention or therapy (like in our case example), but with reproductive choice, the situation is even more complex. Diagnostic tests for fetal aneuploidies, for example, are performed in order to enable the woman to make informed reproductive decisions, without the possibility of primary prevention of the fetal chromosomal condition or of a treatment for it. Decisions in this context resemble in many respects decisions on predictive testing for inherited genetic diseases, where the person at risk is the main decision-maker and beneficence-related criteria only apply in a very limited way. Analogously, it is the private decision-making process of the pregnant woman or of the couple (“Given that we want a child, do we in fact want this specific child?”) which is at the heart of many medical actions in prenatal medicine. It is informed by medical facts but, beyond that, the physician bears only very limited responsibilities. A second decision-making process relates to the health care professionals involved and includes ethical questions regarding the physician’s role, such as “Should we offer a specific kind of prenatal testing, although the test result will be used essentially to inform the pregnant woman and might eventually lead to a termination of this pregnancy?”.

Ethics consultations can have grave implications for both decision-making processes and the proposed course of action. In a Swiss case series on ethics consultation in obstetrics, the decision emerging from the ethics consultations was not to support the wishes of the pregnant women or couples in nine out of 15 cases related to terminations of pregnancy [4]. In a German case series, the recommendation was to decline the request as ethically not justifiable in four out of 13 requests from pregnant women for a late termination of pregnancy [5]. The criteria, on which such recommendations are based, remain largely unclear. There are not only the two potential patients, but in addition very different goals or ends (reproductive choice and possibly the termination of a pregnancy). If enabling reproductive autonomy is at times the primary aim of clinical actions in this field, how can it be justified for an ECS to interfere with the autonomy of the pregnant woman in such a radical way in these cases? Are case-by-case recommendations an adequate response to moral quandaries in relation to a termination of pregnancy in the first place or would elaborate general guidelines be a better way? These are among the questions that should be addressed in order to develop a sound theoretical basis for ethics consultations concerned with reproductive choice. As long as these questions are unanswered, however, the scope for an ECS to contribute to these cases is rather limited.

### Resolving: Conflict mediation in prenatal medicine

In the case of a conflict, an ECS would aim to engage all relevant agents in a dialogue, “where each and every voice is adequately heard and protected” [15] in order to facilitate the building of a consensus between conflict parties. In most prenatal cases the voice of the pregnant woman or couple will without doubt be crucial and should be heard at some point during the process. The critical question, however, is how the voice of the fetus and especially the voice of the potential future child can or should be introduced into the process,—the latter being important even if one understands the fetal interests as a part of the maternal interests.

Even if we do not wish to engage in discussing a right to life for fetuses or embryos, many situations may be imagined where the future child appears to be the addressee of medical actions (e.g. in prenatal therapy) or where its welfare is at least seen as significant. Such circumstances may arise when investigations during the pregnancy may generate information that could harm the interests of the future child or when attempts at treatment may damage the fetus and lead to impairment of the future child. Especially in the light of the ever-growing diagnostic possibilities (for example through fetal whole genome sequencing and/or non-invasive prenatal testing [30]) and increasingly available therapeutic options, which may significantly affect the life of the future child, a facilitation process without any representation of this future child must seem incomplete.

An ECS might argue that this is not an unusual situation. In many cases, the patient is frequently too ill to participate and it is a shared motive of all participants in the process—professionals as well as relatives—to shed light on the wishes and preferences of the patient and “make the patient more than a phantom at the table” [15]. Surrogate decision-makers and advance directives are well-established and helpful institutions in this respect. The difference with the fetus, however, is that the future child has not had a chance to develop wishes and preferences. And the pregnant woman is (usually) a second potential patient who is, at the same time, intimately connected with the fetus, both physically and emotionally. While the couple will have the custody of the future child, the situation of pregnancy cannot be approached with such a concept fixed in advance. The pregnant woman is directly affected by any decision made, so that she might not be able to adopt a position comparable to a surrogate decision-maker for the future child. At the same time, introducing a third party (e.g. a prenatal medicine specialist, an ethicist, a social worker or even a court) as a surrogate for the fetus or the future child would hardly seem to be an acceptable intrusion into the close bodily and emotional connectedness of the pregnant woman and the fetus or future child.

At first sight, the pregnant woman and her partner in our case example can and should participate in a conflict mediation as representatives of their fetus and their future child. The health and the welfare of the fetus and the future child is the main issue at stake. And the planned amniocentesis has only a minor impact on health of the pregnant woman. So the danger of an “inner” conflict of interests seems comparably low for the pregnant woman at this stage. A preparatory meeting of the ECS with the couple, however, could reveal grave concerns regarding their future life with a severely disabled and probably soon dying child, leading to a pregnancy conflict which might contribute to their preference for a palliative treatment.

While the pregnant woman or couple are the natural representatives of the interests of the fetus or the future child in a mediation process, there are situations in which she or they might be overburdened by this role (for example because of an inner “pregnancy” conflict). In such a situation, a joint mediation setting together with the professional team could hardly be successful and in any case an immense stress for the pregnant woman and her partner and should, therefore, be avoided. A series of small meetings with the couple or the pregnant woman alone can, however, clarify the inner conflict and prepare the ground for ethically acceptable conflict solution.

## Conclusion

When concerned with cases in the field of prenatal diagnosis and therapy, it will be the foremost duty of any ECS in prenatal medicine to acknowledge its limitations and to further engage in clarifying and strengthening the theoretical basis of ethics consultation. Pregnancy differs significantly from other fields of action for an ECS. An unreflective application of the general standards of ethics consultation bears significant risks for all parties involved—for the pregnant woman or couple and the fetus or the future child as well as for health care professionals. An ECS working with the facilitation approach should openly communicate the variability and vagueness of ethical concepts in the field of prenatal medicine and avoid content-heavy recommendations,—especially when concerned with cases of reproductive choice. Whenever there are signs indicating a pregnancy-related “inner” conflict of the pregnant woman, small meetings of the ECS with the pregnant woman or couple are an essential first step, before a joint mediation meeting with several parties can be discussed.

Clinicians are important partners in this endeavor of developing appropriate standards for ethics consultation in prenatal medicine. Their practical insight into daily ethical challenges makes them indispensable for the process of working back and forth between principles of professional ethics and moral intuitions in order to elaborate a comprehensive normative framework for this special area in medicine. If the moral intuition of fetal patient-hood, for example, contradicts general principles of moral status and the reproductive rights of the pregnant woman, it might be possible through interdisciplinarity to develop a more adequate alternative. As long as a comprehensive normative framework for prenatal medicine is a desideratum, however, ethics consultation services should proceed with caution and modesty, concentrating on understanding, acting as a sounding board and committed to Socratic dialogue. This would be constructive, useful and, indeed, “ethical”.

## Data Availability

Not applicable.

## Abbreviation

ECS: Ethics consultation service

## Footnotes

1 McCullough and Chervenak, themselves, repeatedly emphasized the moral importance of „clinical links “ between the fetus and the future child [26].

## References

[1] Forde R, Vandvik IH (2005). Clinical ethics, information, and communication: review of 31 cases from a clinical ethics committee. J Med Ethics.

[2] Tapper EB, Vercler CJ, Cruze D, Sexson W (2010). Ethics consultation at a large urban public teaching hospital. Mayo Clinic Proc.

[3] Reiter-Theil S, Schuermann J (2016). The, "Big Five" in 100 clinical ethics consultation cases. Bioeth Forum.

[4] Muggli M, De Geyter C, Reiter-Theil S (2019). Shall parent/patient wishes be fulfilled in any case? A series of 32 ethics consultations: from reproductive medicine to neonatology. BMC Med Ethics.

[5] Wernstedt T, Beckmann MW, Schild RL (2005). Late induced abortion—how to find the best decision. Geburtsh Frauenheilk.

[6] Meyer-Wittkopf M, Spescha P, Cignacco E, Raio L, Surbek DV (2006). Klinisch-ethische Entscheidungsfindungen im Rahmen eines Ethikzirkels bei schwerwiegenden Pränataldiagnostik-Befunden. Geburtsh Frauenheilk.

[7] Thornton JG, Lilford RJ (1995). Clinical ethics committee. BMJ.

[8] Tarzian AJ, ASBH Core Competencies Update Task F (2013). Health care ethics consultation: an update on core competencies and emerging standards from the American Society for Bioethics and Humanities' core competencies update task force. Am J Bioeth AJOB.

[9] Fox E, Danis M, Tarzian AJ, Duke CC (2021). Ethics consultation in U.S. Hospitals: a national follow-up study. Am J Bioeth AJOB.

[10] Fiester A (2015). Neglected ends: clinical ethics consultation and the prospects for closure. Am J Bioeth AJOB.

[11] Siegler M (2019). The ASBH approach to certify clinical ethics consultants is both premature and inadequate. J Clin Ethics.

[12] DeRenzo EG (2019). Moving towards a new hospital model of clinical ethics. J Clin Ethics.

[13] Fiester A (2014). Bioethics mediation and the end of clinical ethics as we know it. Cardozo J Confl Resolut.

[14] Gasparetto A, Jox RJ, Picozzi M (2018). The notion of neutrality in clinical ethics consultation. Philos Ethics Humanit Med PEHM.

[15] Dubler NN, Liebman CB (2011). Bioethics mediation: a guide to shaping shared solutions.

[16] Schmitz D, Gross D, Pauli R (2021). Is there a need for a clear advice? A retrospective comparative analysis of ethics consultations with and without recommendations in a maximum-care university hospital. BMC Med Ethics.

[17] Dondorp WJ, Page-Christiaens GC, de Wert GM (2016). Genomic futures of prenatal screening: ethical reflection. Clin Genet.

[18] Simkulet W (2021). The inconsistency argument: why apparent pro-life inconsistency undermines opposition to induced abortion. J Med Ethics.

[19] Stapleton G, Dondorp W, Schroder-Back P, de Wert G (2019). Just choice: a Danielsian analysis of the aims and scope of prenatal screening for fetal abnormalities. Med Health Care Philos.

[20] Bunnik EM, Kater-Kuipers A, Galjaard RH, de Beaufort ID (2019). Should pregnant women be charged for non-invasive prenatal screening? Implications for reproductive autonomy and equal access. J Med Ethics.

[21] Shakespeare T (2017). A brave new world of bespoke babies?. Am J Bioeth AJOB.

[22] Chervenak FA, McCullough LB (1996). The fetus as a patient: an essential ethical concept for maternal-fetal medicine. J Matern Fetal Med.

[23] Schmitz D, Clarke A, Dondorp W (2018). The fetus as a patient: a contested concept and its normative implications.

[24] Lyerly AD, Little MO, Faden RR (2008). A critique of the 'fetus as patient'. Am J Bioeth AJOB.

[25] Rodrigues HC, van den Berg PP, Duwell M (2013). Dotting the I's and crossing the T's: autonomy and/or beneficence? The 'fetus as a patient' in maternal-fetal surgery. J Med Ethics.

[26] McCullough LB, Chervenak F, Schmitz D, Clarke A, Dondorp W (2018). The ethical concept of the fetus as a patient: responses to its critics. The fetus as a patient: a contested concept and its normative implications.

[27] Smajdor A, Schmitz D, Clarke A, Dondorp W (2018). Means, ends, and the fetal patient. The fetus as a patient: a contested concept and its normative implications.

[28] Premkumar A, Gates E (2016). Rethinking the bioethics of pregnancy: time for a new perspective?. Obstet Gynecol.

[29] Brown SD (2008). The "fetus as patient": a critique. Am J Bioeth AJOB..

[30] Schmitz D, Henn W (2021). The fetus in the age of the genome. Hum Genet.

